# Mast cell activation in the skin of *Plasmodium falciparum* malaria patients

**DOI:** 10.1186/s12936-015-0568-8

**Published:** 2015-02-07

**Authors:** Panop Wilainam, Rungrat Nintasen, Parnpen Viriyavejakul

**Affiliations:** Department of Preclinic and Applied Animal Science, Faculty of Veterinary Science, Mahidol University, Nakhonpathom, 73170 Thailand; Department of Biological Science, Faculty of Science and Technology, Huachiew Chalermprakiet University, 18/18 Bang Na-Trad Road, Km 18, Samut Prakarn, 10540 Thailand; Department of Tropical Pathology, Faculty of Tropical Medicine, Mahidol University, 420/6 Rajvithi Road, Bangkok, 10400 Thailand

**Keywords:** Mast cells, *Plasmodium falciparum*, Malaria, Skin, Toluidine blue stain

## Abstract

**Background:**

Mast cells (MCs) play an important role in the immune response and inflammatory processes. Generally, MCs can be stimulated to degranulate and release histamine upon binding to immunoglobulin E (IgE). In malaria, MCs have been linked to immunoglobulin (Ig) E-anti-malarial antibodies. This study investigated the response of MCs in the skin of patients with *Plasmodium falciparum* malaria.

**Methods:**

Skin tissue samples were examined from ten uncomplicated and 20 complicated *P. falciparum* malaria cases. Normal skin tissues from 29 cases served as controls. Pre- and post-treatment tissues were included. Histopathological changes of the skin were evaluated using haematoxylin and eosin stain. MCs were investigated using toluidine blue staining. The percentage of MC degranulation was compared among groups and correlated with clinical data.

**Results:**

MC degranulation was significantly higher in the complicated *P. falciparum* (43.72% ± 1.44) group than the uncomplicated *P. falciparum* (31.35% ± 3.29) (*p* <0.05) and control groups (18.38% ± 1.75), (*p* <0.0001). MC degranulation correlated significantly with the degree of parasitaemia (*r*_*s*_ = 0.66, *p* <0.0001). Associated pathological features, including extravasation of red blood cells, perivascular oedema and leukocyte infiltration were significantly increased in the malaria groups compared with the control group (all *p* <0.001).

**Conclusions:**

MCs in the skin dermis are activated during malaria infection, and the degree of MC degranulation correlates with parasitaemia and disease severity.

## Background

Malaria is the most serious and widespread parasitic disease in humans, occurring widely throughout the tropical areas of the world. In 2012, 207 million cases of malaria were reported, with 627,000 deaths [1]. The process of malaria pathogenesis is very complex, mainly involving the effects of cytoadhesion and sequestration of parasitized red blood cells (PRBCs) in the vital organs [2] and the induction of soluble cytokines [3,4]. The major complications of severe *Plasmodium falciparum* malaria include cerebral malaria, pulmonary oedema, acute kidney injury, severe anaemia, bleeding, acidosis and hypoglycaemia [5]. These complications can develop rapidly and progress to death within hours or days [6].

Mast cells (MCs) are pivotal effector cells in allergic diseases, and play a role in the body’s inflammatory response and immunity. These immune cells are abundant in tissues exposed to the external environment, including the skin [7,8]. MCs can be activated by various stimuli including cytokines, chemokines and neuropeptides. The biological effects of MCs depend on the release of preformed and *de novo*-synthesized mediators such as histamine, proteases, leukotrienes, and various cytokines [9]. MCs are best known for immunoglobulin (Ig) E-mediated immediate-type hypersensitivity reactions [10]. In *P. falciparum* infection, the significant elevation of the blood concentrations of IgE, IgE-anti-malarial antibodies and histamine have been associated with disease severity in human [11] and animal models [12]. Morphologically, MCs have not been evaluated in malaria. This study aimed to investigate the response of MCs in the skin of patients with *P. falciparum* malaria.

## Methods

### Patients and normal cases

Skin biopsied specimens from *P. falciparum* malaria patients were classified into two groups, uncomplicated (ten cases) and complicated (20 cases) *P. falciparum* malaria cases, according to the available history in the clinical chart, based on WHO criteria [5]. All *P. falciparum* malaria patients enrolled had no history of underlying disease. Normal skin tissues served as control cases (29 cases). All tissues were retrieved from paraffin-embedded specimens collected at the Department of Tropical Pathology, Faculty of Tropical Medicine, Mahidol University. The use of leftover specimens was approved by the Ethics Committee of the Faculty of Tropical Medicine, Mahidol University, Bangkok, Thailand (MUTM 2013-044-01).

### Histopathological study

The collected skin tissues in paraffin blocks were re-embedded, sectioned at 4 μm thickness and stained with routine haematoxylin and eosin (H&E) stain for histopathological examination. MCs were evaluated using toluidine blue stain modified from Mills *et al.* [13]*.* Briefly, after deparaffinization and hydration, the sections were immersed in 0.1% toluidine blue working solution for 5–10 min and washed in distilled water, with three changes. The tissues were then dehydrated quickly through 90%, and two changes of absolute ethanol. Sections were then cleared in xylene, and mounted with mounting medium.

### Evaluation of MCs

MCs (both intact and degranulated forms) were quantified in two areas, the upper dermis (papillary layer) and the lower dermis (reticular and subcutaneous fatty layers). MCs were counted in 15 fields each, at the areas adjacent to the blood vessels, and in the connective tissues, under high-power magnification (400×). For specimens with a small counting area, all MCs were evaluated before the percentage of degranulated MCs was calculated. Parenchymatous changes, including extravasation of RBCs, perivascular oedema, and the presence of leukocytes, were quantified and presented as percentages.

### Statistical analysis

Differences among the three groups were analysed using the Kruskal-Wallis test, and expressed as geometric means ± standard error of the mean (SEM). The Mann–Whitney *U* test was used to compare between malaria groups, and between pre- and post- treatment data. The Statistical Package for the Social Sciences (SPSS) version 11.0 software (SPSS Chicago, IL, USA) was used, and a value of *p* <0.05 was deemed statistically significant.

## Results

### Clinical data of malaria patients

Uncomplicated *P. falciparum* patients usually presented with mild symptoms including fever, headache, myalgia, chills, and nausea. Seventy five percent of complicated malaria cases were associated with cerebral malaria, 30% with acute kidney injury, 45% with hyperparasitaemia, 35% with schizontaemia and 35% with severe anaemia. The clinical details of the malaria patients are shown in Table 1. Variables showing significant differences between the two groups included the number of white blood cells (WBCs), the levels of total and direct bilirubin, aspartate and alanine aminotransferases (all *p* <0.05). Artemisinin derivatives were used for the treatment of both uncomplicated and complicated *P. falciparum* malaria patients.

**Table 1:** **Clinical data of** ***Plasmodium falciparum*** **malaria**

	Uncomplicated* ***P. falciparum*** **malaria (** ***n = 5*** **)**	**Complicated** ***P. falciparum*** **malaria (** ***n = 20*** **)**
Age	28	29
Gender (male: female)	5:0	12:8
Days of fever (*p* = 0.41)	3.6 ± 0.98	4.45 ± 0.44
Admission parasite count/μL (*p* = 0.51)	52,638 ± 12,990	181,315 ± 55,314
WBC (×10^9^/L) (*p* = 0.041)	5.82 ± 1.24	9.61 ± 0.79
Hb (g/dL) (*p* = 0.17)	12.70 ± 1.35	11.10 ± 0.56
Platelets (×10^9^/L) (*p* = 0.20)	50.60 ± 7.98	70.85 ± 28.69
BUN (ml/dL) (*p* = 0.51)	24.84 ± 4.86	34.96 ± 5.70
Creatinine (ml/dL) (*p* = 0.73)	1.06 ± 0.11	2.21 ± 0.70
TB (mg/dL) (*p* = 0.01)	1.91 ± 0.34	5.88 ± 1.14
DB (mg/dL) (*p* = 0.0073)	0.56 ± 0.23	3.34 ± 0.80
AST (IU/L) (*p* = 0.0012)	25.40 ± 3.56	112.0 ± 21.50
ALT (IU/L) (*p* = 0.0039)	21.80 ± 5.23	90.85 ± 15.19

*Clinical data available for 5 uncomplicated *P. falciparum* malaria patients.

WBC-white blood cell count, Hb-haemoglobin, BUN-blood urea nitrogen, TB-total bilirubin, DB-direct bilirubin, AST-aspartate aminotransferase, ALT-alanine aminotransferase. Data expressed as mean ± SEM.

### Evaluation of degranulated mast cells

**Figure 1:**
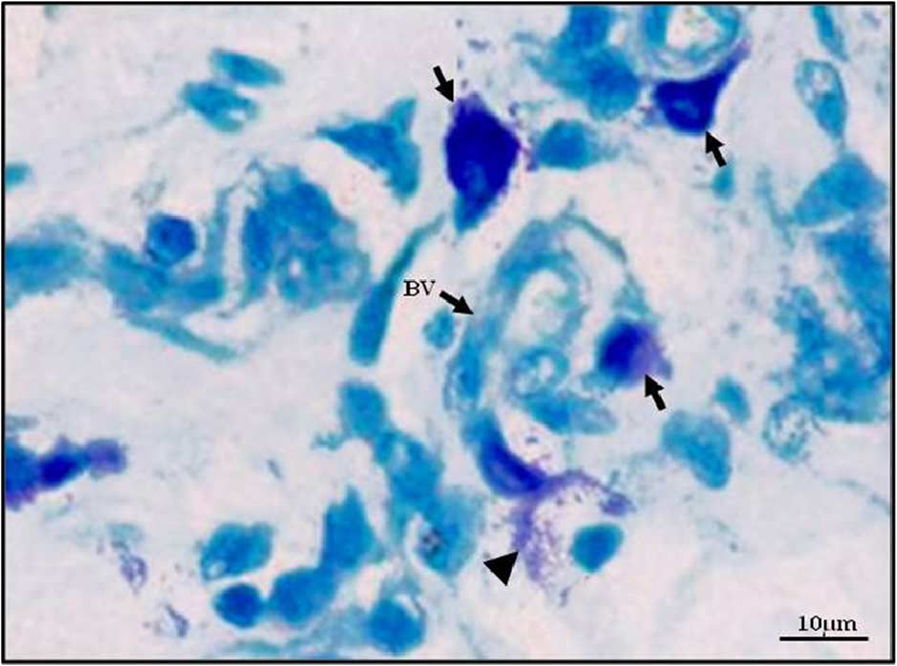
**Intact (arrow) and degranulated (arrow head) MCs around a small blood vessel in the dermis.** BV-blood vessel, toluidine blue stain, 400× magnification, Bar = 10 μm.

**Figure 2:**
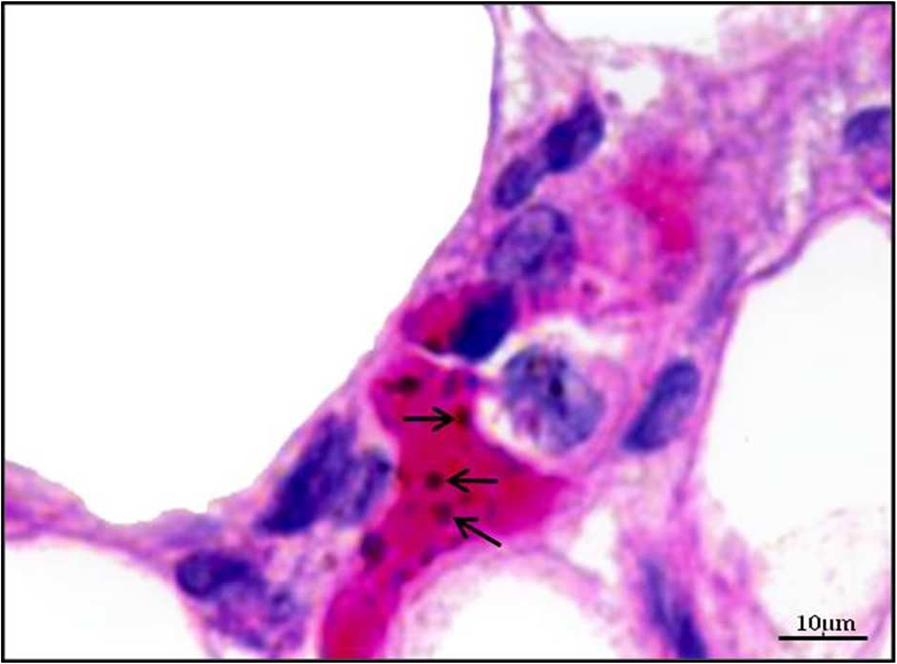
**Parasitized red blood cells (arrows) within the dermal blood vessel.** Haematoxylin and eosin stain, 400× magnification, Bar = 10 μm.

**Figure 3:**
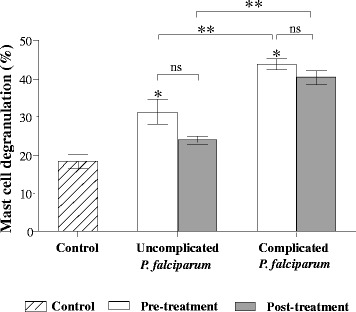
**Percentage MC degranulation in** ***Plasmodium falciparum*** **malaria.** MC degranulation in malaria patients was significantly increased compared with controls. *p <0.05- significant difference as compared to control group, **p <0.05- significant difference between uncomplicated and complicated malaria groups, ns- non-significant.

**Figure 4:**
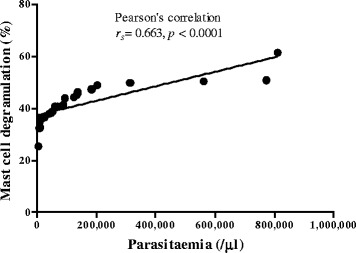
**Correlation between percentage MC degranulation and parasitaemia (/μl).**

An intact MC is defined as a MC with granulated structures confined within the cell membrane, while a MC is considered degranulated when granules are found outside the cell (Figure 1). PRBCs were occasionally seen in the dermal blood vessels (Figure 2). MC degranulation was significantly higher in the complicated *P. falciparum* (43.72% ± 1.44) group than the uncomplicated *P. falciparum* (31.35% ± 3.29, *p* <0.05) or control group (18.38% ± 1.75, *p* <0.0001) (Figure 3). In addition, MC degranulation in the uncomplicated *P. falciparum* malaria cases was significantly higher than the control group (*p* <0.0001). No difference was found between pre- and post-treatment in both malaria groups. The mean number of MCs per high power field (HPF) was 2.54 ± 0.10 in the control group, 1.83 ± 0.24 in the uncomplicated *P. falciparum* group, and 2.69 ± 0.22 in the complicated *P. falciparum* group. At seven days post-treatment, total percentage MC degranulation remained elevated in both malaria groups, similar to pre-treatment (*p* >0.05). MC degranulation percentage correlated significantly with parasitaemia (*r*_*s*_ = 0.66, *p* <0.0001) (Figure 4).

### Histopathologic changes in the skin of malaria patients

**Figure 5:**
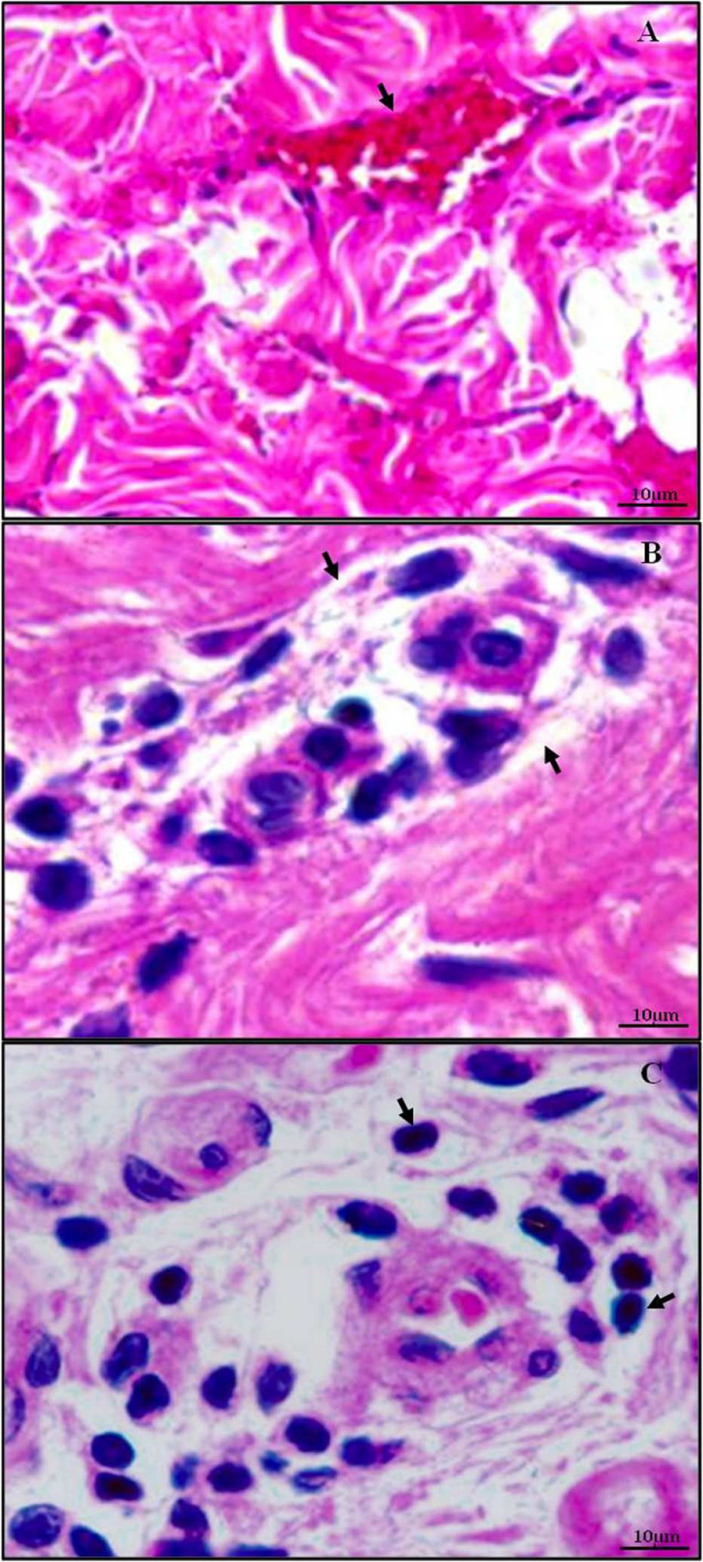
**Histopathologic findings of the skin in** ***Plasmodium falciparum*** **malaria patients.** Extravasation of RBCs in the dermis is shown in **(A)**. **(B)** illustrates perivascular oedema, and **(C)** shows leukocytic infiltration around blood vessels. Haematoxylin and eosin stain, 400× magnification, Bar = 10 μm.

**Figure 6:**
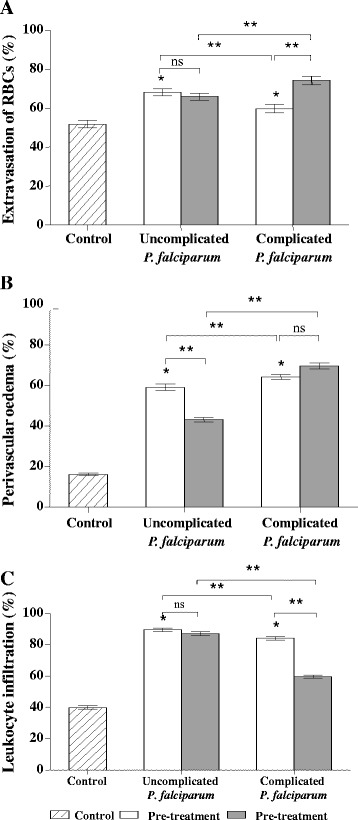
**Comparative histopathologic changes in the skin of** ***Plasmodium falciparum*** **malaria patients.** All histopathologic changes were significantly increased in malaria patients compared with controls. **(A)** Percentage extravasation of RBCs, **(B)** Perivascular oedema, and **(C)** Leukocyte infiltration, *p <0.05- significant difference as compared to control group, **p <0.05- significant difference between uncomplicated and complicated malaria groups, or between pre- and post-treatment groups, ns- non-significant.

The skin epidermis of all groups appeared similar and unremarkable. Within the dermis, RBC extravasation, perivascular oedema, and leukocytic infiltration were significant features of complicated *P. falciparum* malaria (Figure 5). Extravasation of RBCs or diapedesis is defined as the presence of RBCs intermixed with PRBCs and leukocytes within the skin parenchyma [14] (Figure 5A). Extravasated RBCs were more commonly seen in the papillary layer, and significantly more numerous in *P. falciparum* malaria than control cases (all *p* <0.05)*.* In complicated *P. falciparum* malaria, RBC extravasation was significantly increased (by 23.76%) in post-treatment, compared with pre-treatment (*p* <0.0001). However, there was no change in RBC extravasation between pre- and post-treatment in the uncomplicated malaria group (Figure 6A). Perivascular oedema is determined by the presence of perivascular clearing due to unstained fluid accumulation around blood vessels (Figure 5B). This is commonly found around small blood vessels deep in the dermis, in the reticular and subcutaneous fatty layer more than in the papillary layer. The perivascular oedema of uncomplicated and complicated *P. falciparum* malaria was significantly higher than control group (all *p* <0.001). In the post-treatment group, perivascular oedema was significantly decreased by 36.05% in uncomplicated *P. falciparum* malaria, compared with pre-treatment (*p* <0.0001); however, this was not observed in complicated *P. falciparum* malaria (Figure 6B). Commonly observed leukocyte infiltrations included mononuclear cells, such as lymphocytes and monocytes; while neutrophils, eosinophils, basophils, and plasma cells were rare. Leukocytes appeared to infiltrate around the blood vessels and were located mostly in the reticular layer (Figure 5C). Leukocytic infiltration in malaria groups was significantly higher in both uncomplicated and complicated *P. falciparum* malaria compared with the controls (all *p* <0.0001) (Figure 6C). Leukocytic infiltration decreased by 28.61% in complicated *P. falciparum* post-treatment (*p* <0.0001), but there was no change in uncomplicated *P. falciparum* malaria*.*

## Discussion

MCs have been implicated in the inflammatory response and allergic process by releasing various cytokines, associated with IgE-mediated immediate-type hypersensitivity reactions [9,15]. This study is the first to investigate the response of MCs in the skin tissues of *P. falciparum* malaria-infected patients. MCs were localized mostly in the papillary layer, which correlated with blood vessel density and contributed to an immediate defense against stimuli [16]. MC degranulation is a characteristic pattern of immunological and morphological changes after activation [17]. Generally, an increase in degranulated MCs can result in MC activation syndrome (MCAS). The syndrome includes a range of manifestations, involving the skin, gastrointestinal, cardiovascular, respiratory, and neurological system [18]. In malaria infection, studies have shown that IgE level is associated with malaria severity [19,20]. In addition, malaria antigens have been reported to activate macrophages and monocytes to produce various cytokines, such as tumour necrosis factor (TNF)-alpha [20,21] and interleukin-1 beta [22]. These cytokines, which are classified as MC secretagogues, may activate MCs through FcεRI receptors. The increased level of these cytokines is related to malaria severity, and is hypothesized to affect the response of MCs. MCs provide a potential source of TNF during the early phase of infection. In an animal malaria model, a study showed an association between MCs and the elevation of serum TNF, which could contribute to malaria protection [23]. Increases in the number of MCs, and MC degranulation, have been documented in diseases such as renal amyloidosis [24], interstitial lung fibrosis [25], gastrointestinal disease [26] and myocardial infarction [27]. The changes correlated with pathological alterations, and are relevant to MC mediators.

Artemisinin derivatives were the drugs of choice used to treat all malaria cases. A previous report has documented that artemisinin can decrease MC degranulation by blocking IgE-induced MC degranulation in an anaphylactic animal model [16]. However, in this study, the number of MC degranulations remained elevated at seven days post-treatment. Thus, artemisinin may not prevent MC degranulation in malaria *in vivo*. Quinine and tetracycline have also been reported to cause MC degranulation [28]. Mefloquine was recently reported to function as an anti-mast cell agent, inducing apoptosis through a granule-mediated pathway [29]. The effects of anti-malarial drugs on mast cell degranulation need to be further investigated to determine the physiological action and pathways involved. Moreover, some antibiotics, including penicillins, cephalosporins, sulphonamides, as well as some antiepileptic drugs can cause MC degranulation by acting through IgE receptors on MCs, triggering degranulation [28].

Changes in the number of MCs have been reported in wound healing [30], keloid formation [31], chronic inflammation [32], parasitic infestation [33], urticaria, atopic eczema, lichen planus, psoriasis, pretibial myxedema, scleroderma, neural tumour and mycosis fungoides [17]. The factors involved in the increased number of MCs in such conditions remain unclear [34]. In addition, percentage MC degranulation correlated positively with initial parasite count (*r*_*s*_ = 0.66, *p* <0.0001), indicating that MCs are activated in severe *P. falciparum* malaria. Normally, MCs can undergo repeated rounds of degranulation and regranulation [35-38]. An *in vitro* human lung MC culture showed late recovery (18–48 hr) from IgE-induced MC degranulation [39]. In addition, mouse MCs recovered from degranulation at 30 min after activation [40]. However, another study reported MCs regenerated from horseradish peroxidase induced MC degranulation after six weeks [41]. At day 7 post-treatment, MC degranulation of uncomplicated (day 0 = 31.35% ± 3.29, day 7 = 23.93% ± 1.07, *p* = 0.091) and complicated *P. falciparum* (day 0 = 43.72% ± 1.44, day 7 = 40.32 ± 1.81%, *p* = 0.349) remained elevated. The process of MC regranulation at day 7 was not observed in the study, indicating that MC regranulation in the skin of *P. falciparum* is a delayed response (over seven days).

Parenchymatous changes, including RBC extravasation, perivascular oedema and leukocytic infiltration were significantly increased in the malaria groups. It can be speculated that RBC extravasation and perivascular oedema are secondary to endothelial cell damage, following cyto-adhesion and sequestration of PRBCs, and the local effects of MC cytoplasmic granules, such as TNF, histamine, heparin, and proteases [42]. In addition, histamine, the major MC mediator in malaria, causes increased vascular permeability and subsequent extensive vascular damage to endothelial cells [43]. Inflammatory reactions can also be related to immune responses during malaria infection.

## Conclusions

MC activation and degranulation are observed in the skin dermis of severe *P. falciparum* malaria patients. In addition, the number of MCs and the degree of degranulation correlate with parasitaemia and disease severity. Associated histopathological changes in the dermis during malaria infection include extravasation of RBCs, perivascular oedema and leukocyte infiltration around the blood vessels.
